# Urinary Incontinence Following Robotic-Assisted Radical Prostatectomy: A Literature Review

**DOI:** 10.7759/cureus.53058

**Published:** 2024-01-27

**Authors:** Hamzeh Farraj, Sulieman Alriyalat

**Affiliations:** 1 Department of Special Surgery, Division of Urology, Al-Balqa Applied University, Salt, JOR

**Keywords:** post-surgical outcomes, literature review, urinary incontinence, prostate cancer, robot-assisted radical prostatectomy

## Abstract

Prostate cancer ranks as one of the most prevalent cancers among men in the United States, contributing significantly to cancer-related mortality. Robot-assisted radical prostatectomy (RARP) has become a cornerstone in the management of localized prostate cancer.

This literature review delves into the outcomes of RARP, specifically its impact on urinary incontinence (UI) compared to other surgical methods. We also present the importance of patient perception versus medical reports.

Recent studies and trials have unveiled that postoperative UI and erectile dysfunction (ED) remain common concerns following prostatectomy. However, studies have shown that RARP has lower occurrences of UI and ED compared to radical retropubic prostatectomy (RRP).

While the choice of surgical method may not drastically affect these outcomes, the review emphasizes that urinary incontinence extends beyond physical symptoms. It profoundly impacts patients' psychological well-being, social interactions, and overall quality of life. Differences in symptom recording and interpretation between patients and healthcare professionals can significantly influence the diagnosis and treatment of prostate cancer. Enhanced patient-physician communication and patient-centered care are essential to providing a holistic approach to prostate cancer management.

The choice of surgical methods may not significantly impact postoperative urinary incontinence and erectile dysfunction. Continued research and advancements in treatment and patient care are crucial for improving outcomes and the overall well-being of prostate cancer patients.

## Introduction and background

Prostate cancer ranks as one of the most prevalent cancers among men in the United States, in addition to non-melanoma skin cancer. Furthermore, it stands as a significant contributor to cancer-related mortality among men of all racial and Hispanic origin groups [1]. It is estimated that the incidence of new prostate cancer cases in 2023 will reach 288,300 cases, accounting for 15% of all new cancer cases [2]. The highest rates of prostate cancer are shown to be associated with black, non-Hispanic groups with an age-adjusted ratio of 154.7 and among age groups between 70 and 74 years [3]. The five-year survival rate of prostate cancer reached 97.1% in the years between 2013 and 2019, with the relative survival rate for black men being lower than that for white men [4].

According to the European Association of Urology (EAU), the European Association of Nuclear Medicine (EANM), the European Society for Radiotherapy and Oncology (ESTRO), the European Society of Urogenital Radiology (ESUR), and the International Society of Geriatric Oncology (SIOG) guidelines on the management and treatment of prostate cancer, radical prostatectomy (RP), external beam radiotherapy (EBRT), and active surveillance are the main treatment options for localized prostate cancer that have demonstrated comparable results in terms of overall survival (OS) and cancer-specific survival (CSS). The treatment option is usually based on the patient's preference and risk stratification. Watchful waiting is an option for patients with localized prostate cancer who have a life expectancy of less than ten years [5,6].

Robot-assisted radical prostatectomy (RARP) is one of the surgical approaches in RP that is associated with decreased perioperative complications and a lower incidence of positive surgical margins in contrast to laparoscopic prostatectomy, although there remains a degree of methodological uncertainty [7,8]. Postoperative incontinence (involuntary leakage of urine) and erectile dysfunction (ED; defined as the recurrent inability to achieve or maintain an erection) are prevalent issues that can arise after RP. Notably, there is little discrepancy based on the surgical approach, as the overall continence rate ranges from 89% to 100% when a robotic procedure is performed, compared to a range of 80% to 97% in a retropubic approach [9,10].

Thus, in this review article, we aimed to assess the outcomes of RARP in terms of urinary incontinence (UI) compared to other surgical approaches.

## Review

Statistics and previous studies (RCTs) on AEs of RARP

Surgical treatments for prostate cancer are not free of adverse events. Recent systematic reviews of observational studies have hinted that RARP may result in lower occurrences of urinary incontinence compared to laparoscopic radical prostatectomy (LRP), similar to retropubic radical prostatectomy (RRP), and a higher occurrence compared to brachytherapy (BT) [9]. The ProtecT, which is a randomized controlled trial (RCT) by Barry et al. comparing urinary incontinence between RARP and RRP on a nationwide sample of Medicare-age men, showed that 31% had problems with continence, varying from mild to severe stress urinary incontinence (SUI), and 88% had problems with sexual dysfunction. However, there were no significant differences or associations between urinary incontinence or sexual dysfunction and RARP [11]. In the phase III trial by Yaxley et al., there were no significant differences in urinary incontinence or sexual dysfunction between RARP and RRP, suggesting these two surgical approaches yield similar outcomes [12]. Similarly, a large population-based study by Fridriksson et al. showed that the likelihood of experiencing urinary incontinence was comparable for both surgical methods when evaluating diagnostic and intervention codes [13].

Efficacy of new treatments

Managing urinary incontinence after prostatectomy remains a complex issue for both urologists and patients following prostate surgery. While urinary incontinence can develop after interventions for benign prostatic enlargement, the most distressing and life-altering form is stress urinary incontinence, which affects men after undergoing radical prostatectomy and other treatments for prostate malignancies [14].

Adjustable continence therapy is a procedure for men known as ProACT that involves the insertion of two silicone balloons through a perineal approach. This procedure can utilize either endoscopic assistance, real-time fluoroscopy, or transrectal ultrasound guidance [15]. Titanium ports are affixed to each balloon, and they can be accessed through the scrotum, enabling straightforward adjustments to the balloon volume [16,17]. The utilization of ProACT remains limited among men dealing with urinary incontinence.

Although there were previous efforts to create artificial sphincters before the 1970s, the credit for designing and publishing the first hydraulic artificial urinary sphincter (AUS) goes to Scott et al., who introduced the AS721 devices in their 1973 publication [18]. In 2015, the consensus statement from the International Continence Society recommended that men with persistent SUI lasting more than six months should be provided with treatment options. It also suggested that men with sufficient manual dexterity should be considered for the AUS when suitable. Delaying treatment beyond the six-month mark is unlikely to improve the outcomes for patients experiencing incontinence after undergoing RRP [19,20]. Infections involving the AUS device can manifest either shortly after the surgery or several years following its implantation. Early infections tend to be more frequent and often necessitate the removal of the device [21].

Patient perception vs. medical reports

The impact of urinary incontinence extends beyond its substantial socio-economic burdens on both patients and society, encompassing adverse effects on patients' quality of life, including stigmatization, social isolation, depressive symptoms, loneliness, and embarrassment [22]. A study by Chow et al. showed that stress urinary incontinence is an independent risk factor for anxiety and depression and has been linked to significant work dysfunctions [23].

Discrepancies in symptom recognition

Inconsistencies and differences in the way symptoms are identified and reported between healthcare professionals and patients can occur when comparing the objective assessment of symptoms by medical practitioners with the subjective experience and reporting of those symptoms by patients [24,25]. It is well known that the documentation of symptoms and clinical signs can exhibit variation across medical practices. Physicians might provide a more comprehensive symptom recording if they suspect the presence of cancer. In such cases, it is possible that the positive predictive values have been overestimated. Conversely, the scenario of increased symptom documentation when no diagnosis is evident but less probable [26,27].

Enhancing patient-centered care

Patient-centered care involves delivering healthcare that acknowledges and is adaptable to the personal preferences, requirements, and principles of each patient, with patient values serving as the cornerstone for all decision-making [28]. Physician treatment recommendations serve as significant indicators of the treatment choices patients make. Hence, it is crucial to ascertain the extent to which physicians accurately align their perspectives on preferences with those of the patients [29]. An online tool was developed by Jayadevappa et al. called PreProCare, designed for assessing preferences in prostate cancer treatment, and aims to assist recently diagnosed localized prostate cancer patients in comprehending their own preferences. In doing so, it promotes informed decision-making regarding their treatment options [30]. The significance of the healthcare role in delivering specialized supportive and clinical care is well acknowledged. A study by Reynolds et al. showed that aligning with patients' viewpoints underscored the substantial advantages derived from the interpersonal and technical expertise of specialized clinical and continence nurses [31-33]. This not only fostered patient advocacy but also enhanced readiness for surgery and increased adherence to pelvic floor strengthening measures [34].

Quality of life post-prostatectomy

Following a radical prostatectomy, mental health challenges often correlate with urinary and sexual issues, specifically urinary incontinence, erectile dysfunction, and sexual impotence. These issues can lead to symptoms of depression and a decline in the overall quality of life one year post-surgery [35-36].

Psychological toll of urinary incontinence

It is inevitable that all types of incontinence carry psychological repercussions, with feelings of shame and insecurity frequently stemming from the uncontrolled loss of urine. Over time, these emotions can contribute to avoiding social interactions and potentially result in depression and isolation, among other consequences [37]. A study by Anguas-Gracia et al. assessed the quality of life before and after radical prostatectomy using the prostate cancer-specific module of the EORTC QLQ-C30 questionnaire [38], showing that following radical prostatectomy, patients exhibited lower scores in areas related to role functioning, social engagement, and financial well-being [35]. In addition, a meta-analysis on social well-being after radical prostatectomy showed that the physical symptoms experienced after the surgery also induced a substantial fear of being stigmatized. Urinary incontinence necessitated the use of protective pads to prevent leaks, which, in turn, led to feelings of shame and embarrassment in social situations [39]. Following RARP, a considerable number of patients undergo moderate-to-severe urinary incontinence, reaching as high as 74.3% [40]. However, these rates gradually decrease to 46.8% after 1 month, 21.4% after 3 months, 13.6% after 6 months, and 9% after 12 months [41,42]. A study conducted by Carrier et al. revealed that RARP has an adverse effect on men's quality of life, leaving them feeling unprepared. Adequate physical and psychosocial support is deemed essential in such cases [43].

Social implications

While incontinence may not be a life-threatening condition, the loss of bladder control can profoundly impact various facets of patients' lives, including their social, psychological, familial, occupational, physical, and sexual well-being [44]. When comparing RARP to open radical prostatectomy, there were no significant differences in quality of life between the two approaches in the study by Wallerstedt et al. post-operatively. However, pre-operatively, the quality of life of patients who underwent open radical prostatectomy was significantly lower compared to those who underwent RARP. Moreover, the presence of urinary incontinence and erectile dysfunction worsens the quality of life [45].

Physical challenges and quality of life 

Eight studies on the physical implications of prostate cancer survivors following prostatectomy showed that post-surgery, men encountered numerous physical obstacles throughout their healing process, which had significant ramifications for their social welfare [46-53]. A cross-sectional study by Ashton et al. assessed the physical characteristics of post-RARP patients, showing that a subset of men faces an elevated risk of cardiovascular disease within 10 years following a RARP and experiences significant fatigue. As a result, clinicians should take these factors into account when advising patients regarding RARP [54]. Fatigue in cancer patients and cancer survivors has been linked to decreased levels of physical activity, which may have a potential negative impact on their cardiovascular health and hinder their recovery to achieve full functional fitness following RARP [55,56]. Another study by Ashton et al. showed that resistance exercise training can be a beneficial approach to quality of life and muscular strength in patients who underwent RARP [57]. Numerous investigations have examined various time intervals following prostatectomy to evaluate quality of life parameters. However, limited information is available regarding how the choice of surgical procedure, whether open or robot-assisted, affects the duration patients need for social recovery [58]. In the study by Bier et al., they showed that there were no significant differences in the quality-of-life parameters related to social life, daily activities, or return to daily life between patients who underwent open proctectomy and RARP [59].

## Conclusions

Prostate cancer, a pressing public health concern, demands a thorough exploration of its management. Radical prostatectomy, particularly the robot-assisted approach (RARP), emerges as pivotal, offering enhanced overall and cancer-specific survival. This review accentuates RARP's impact on postoperative quality of life, particularly urinary incontinence outcomes. Despite persistent concerns like urinary incontinence and erectile dysfunction post-prostatectomy, the choice of surgical method minimally influences these outcomes. Patient-centered care, encompassing innovations like ProACT and traditional options, aims to address challenges and improve postoperative quality of life.

In emphasizing the interplay between patient perception and medical reports, the review underscores the importance of enhanced patient-physician communication. This approach is critical for a holistic management strategy, acknowledging the broader impact of prostate cancer on psychological and social well-being. As advancements continue, the review highlights the importance of two of the most important postoperative complications when treating patients with localized disease by robotic radical prostatectomy and advocates for ongoing research to refine outcomes and overall well-being for prostate cancer patients.
